# Microcontroller Unit-Based Wireless Sensor Network Nodes: A Review

**DOI:** 10.3390/s22228937

**Published:** 2022-11-18

**Authors:** Ala’ Khalifeh, Felix Mazunga, Action Nechibvute, Benny Munyaradzi Nyambo

**Affiliations:** 1 School of Electrical Engineering and Information Technology, German Jordanian University, P.O. Box 35247, Amman 11180, Jordan; 2 Applied Physics and Telecommunications Department, Midlands State University, Gweru P. Bag 9055, Zimbabwe; 3 Faculty of Technology, Zimbabwe Open University, Harare P.O. Box 8306, Zimbabwe

**Keywords:** Internet-of-Things (IoT), wireless sensor networks, sensor node, microcontroller unit (MCU), power consumption

## Abstract

In this paper, a detailed review of microcontroller unit (MCU)-based wireless sensor node platforms from recently published research articles is presented. Despite numerous research efforts in the fast-growing field of wireless sensor devices, energy consumption remains a challenge that limits the lifetime of wireless sensor networks (WSNs). The Internet-of-Things (IoT) technology utilizes WSNs for providing an efficient sensing and communication infrastructure. Thus, a comparison of the existing wireless sensor nodes is crucial. Of particular interest are the advances in the recent MCU-based wireless sensor node platforms, which have become diverse and fairly advanced in relation to the currently available commercial WSN platforms. The recent wireless sensor nodes are compared with commercially available motes. The commercially available motes are selected based on a number of criteria including popularity, published results, interesting characteristics and features. Of particular interest is to understand the trajectory of development of these devices and the technologies so as to inform the research and application directions. The comparison is mainly based on processing and memory specifications, communication capabilities, power supply and consumption, sensor support, potential applications, node programming and hardware security. This paper attempts to provide a clear picture of the progress being made towards the design of autonomous wireless sensor nodes to avoid redundancy in research by industry and academia. This paper is expected to assist developers of wireless sensor nodes to produce improved designs that outperform the existing motes. Besides, this paper will guide researchers and potential users to easily make the best choice of a mote that best suits their specific application scenarios. A discussion on the wireless sensor node platforms is provided, and challenges and future research directions are also outlined.

## 1. Introduction

Of late, there have been numerous research efforts in the fast-growing field of wireless sensor devices. This is being driven by the advent of many Internet-of-Things (IoT) applications and scenarios that utilize wireless sensor networks (WSNs) for providing an efficient sensing and communication infrastructure. A wireless sensor network consists of distributed wireless sensor nodes or motes that can be used for detecting various physical and environmental phenomena. The nodes collaboratively send the sensed data to a base station or sink for further processing and decision-making. Examples of sensed parameters include temperature, pressure, moisture, motion, liquid level, etc. Wireless sensor networks are very useful in a wide range of applications that include environmental monitoring, precision agriculture, IoT, water quality monitoring, animal tracking and so forth [1,2,3,4]. Overas the years, several practical wireless sensor nodes or motes have been developed and introduced in the market to facilitate the implementation of various wireless sensor network scenarios. Advancements in wireless sensor node technologies [5] facilitate the cutting-edge research and real-world implementation of WSNs in countless applications around the globe.

A sensor node typically consists of the sensing, computation, communication and power supply sub-systems. The wireless sensor node platforms are resource-constrained when it comes to the power supply, computation and storage capabilities, etc. [6]. Some of the objectives of designing and developing a wireless sensor node include ultra-low- power operation, low cost per node, small size and reconfigurable software and hardware. The designing of increasingly smaller and more affordable wireless sensor node platforms of ultra-low power consumption has become a hot research topic for the research community [7].

The lifetime of a WSN strongly depends on the supply of energy for powering the sensor motes. Traditionally, sensor nodes are powered by small batteries of limited capacities. In numerous application scenarios, many deployed sensor motes are required to last unattended for an unlimited lifetime in remote and harsh fields. In such deployment scenarios, battery replacement becomes a big challenge. The energy consumption of the motes directly impacts the lifetime of the WSNs. Therefore, ultra-low power approaches together with energy-harvesting techniques are critical towards the ubiquitous and perpetual operation of WSNs [8]. Due to the different requirements of various WSN applications, different wireless sensor node platforms are being developed. For instance, some tracking and monitoring application scenarios of WSNs require the use of wearables.

Over the years, several wireless sensor nodes based on the MCU, field programmable gate arrays (FPGAs), system-on-chip (SoC), application-specific integrated circuit (ASIC) and other platforms have been designed and developed [9]. In the literature, there is a limited number of review articles on wireless sensor node platforms. In 2009, [10] presented a comparative review of only six selected sensor motes. Authors in [2] provided a survey on the protocols, platforms and simulation tools of selected wireless sensor platforms. A comparison of the performance of two commercial motes in practical situations was performed by [11]. A review of sensor motes targeted only for IoT applications was presented in 2018 by [12]. However, in this proposed study, the selected wireless sensor nodes are used for a wide range of applications. Furthermore, the authors of [9] provided a detailed review on wireless sensor nodes spanning from simple MCU-based to complex programmable logic devices. 

In the literature, there are several other published articles that list and compare different motes that can be used for different IoT applications [13,14,15,16,17]. However, these articles do not include the motes that have been recently proposed and published in the literature. Furthermore, the cited articles do not have detailed comparisons and descriptions of these motes, while this paper lists the most recent motes published in the literature and provides more comparison details, which makes it very valuable to IoT application developers and solution providers. 

In this paper, we concentrate on providing a detailed comparative review of MCU-based wireless sensor nodes from recently published research articles from the year 2016 to 2022. The sensor nodes from the recent research articles are compared with the most widely used commercially available motes based on processing and memory specifications, communication capabilities, power supply and consumption, sensor support, potential applications, node programming, hardware security and so on. This paper seeks to provide a clear picture of the trend towards the design of energy-efficient and autonomous wireless sensor nodes to avoid redundancy in research by industry and research institutions. Of particular interest is to understand the trajectory of development of these devices and the technologies, so as to inform research and application directions. This review paper is expected to stimulate developers of wireless sensor nodes to propose improved platforms that outperform the existing motes. Besides, we also anticipate this paper to guide researchers and potential users to easily make the best choice of a sensor mote that best matches their specific application scenarios. The rest of the paper is organized as follows. Section 2 provides an overview of the wireless sensor node’s main components. Section 3 summarizes the recent MCU-based hardware platforms and the popular commercial motes available in the market. Section 4 provides a technical discussion on the surveyed MCU-based platforms. Finally, Section 5 concludes the paper and proposes future work.

## 2. Wireless Sensor Node: An Overview

**Figure 1:**
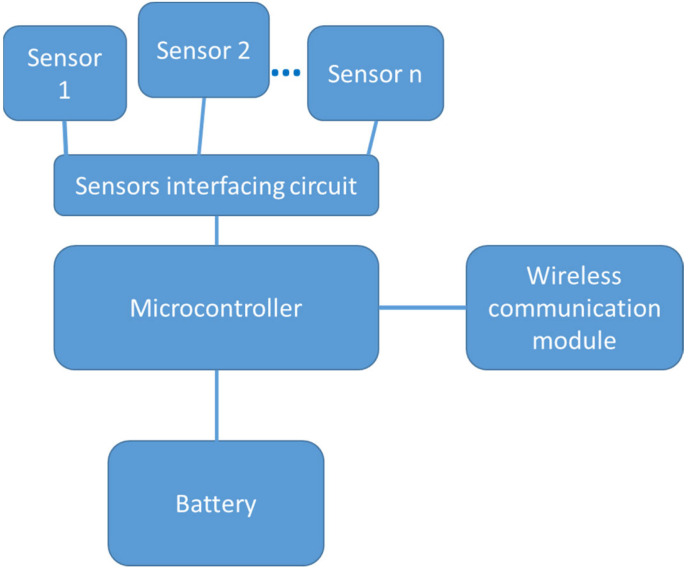
Basic architecture of a sensor node [13].

Although several wireless sensor node platforms based on the MCU have been proposed, they generally have the same basic architecture. They differ with regard to processing and memory specifications, communication capabilities, power supply and consumption, sensor support, applications and so on. A basic wireless sensor node consists of the sensing (sensors interfacing and sensors), computation (a microcontroller), communication and power supply sub-systems as depicted in Figure 1. 

### 2.1. Sensing Sub-System

Sensors convert physical phenomena into electrical signals [5]. Therefore, the sensing sub-system links the node to the environment. Digital or analogue signals are obtainable from the sensors. The sensors’ interfacing circuit enables the microcontroller to process the detected signals. Some of the existing node platforms have embedded or built-in sensors while others do not. Several sensor nodes have sockets capable of accommodating a variety of sensors to be connected thus provisioning for flexibility. 

### 2.2. Computation Sub-System

The tasks of the microcontroller include the processing of the signals coming from the sensing sub-system and transceiver, and coordinating the activities of other sensor mote components [14]. The major goal of the central processing unit (CPU) core is to ensure correct program execution. Thus, the CPU should have the ability to perform tasks such as accessing memories, performing computations, coordinating external devices and interrupt handling. Processors in sensor motes are capable of functioning in different operational modes such as active, idle and sleep modes as the least to mitigate unnecessary power consumption. The storage section of the mote normally comprises the flash memory and random access memory (RAM). The flash memory stores the program code for the sensor mote and facilitates high speed sampling and flexible program updates while the RAM is utilized for the storage of sensed information and any data required for performing computations [15]. The electrically erasable programmable read-only memory (EEPROM) can be used for the storage of the information for identifying the registered sensor motes in the network [16]. Some of the motes have an embedded micro–secure digital (SD) card interface to provide the mote with a non-volatile memory for off-line data capturing. However, the process of writing to the SD card is power-demanding.

### 2.3. Communication Sub-System

The communication subsystem comprises a radio module or transceiver for facilitating the reception and transmission of signals to and from the wireless sensor motes, and also to a base station or sink [14]. Radio frequency (RF) communication is suitable for WSNs because it is not limited by line-of-sight (LOS) and recent advances allow the utilization of low-power radio modules with data rates and communication ranges adjustable depending on the application scenarios.

### 2.4. Power Supply Sub-System

The power supply sub-system usually consists of a battery and/or a supercapacitor for storing and powering the sensor node. For the different application scenarios of wireless sensor networks, the intended lifetime of the sensor nodes may be several years, and battery replenishment or replacement can be costly, inconvenient or even impossible, especially in large-scale, remote and hazardous environments. Therefore, designers introduce ultra-low-power and energy-efficient sensor nodes including the associated communication protocols and the motes’ sleep/awake mode schedules. Energy harvesting from potential sources such as RF signals, solar, vibrations and so on may also be incorporated to recharge the batteries [17]. 

## 3. MCU-Based Hardware Platforms for Wireless Sensor Nodes

**Figure 2:**
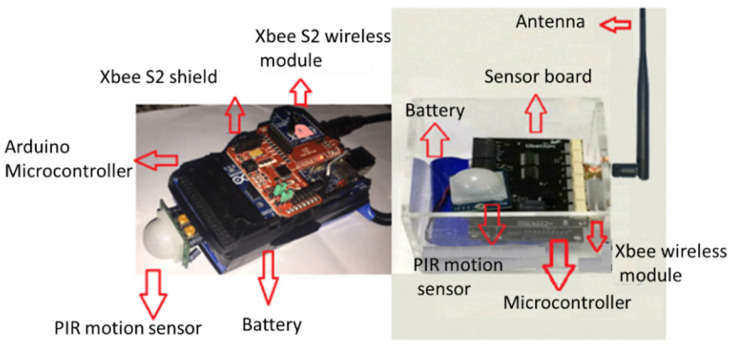
Images of two typical MCU-based wireless sensor nodes [18].

This section provides a review of wireless sensor nodes from recently published research articles and the popular commercially available motes with respect to their potential applications, processor and memory specifications, communication capabilities, sensor support, power supply and power consumption. The sensor nodes from recent research works were selected from internationally recognized databases. The commercially available motes (MICAz, IRIS, LOTUS, WiSense and Waspmote Plug & Sense!) were selected for comparison in this study based on a number of criteria including popularity, published results and interesting characteristics and features. The popular MiCAz mote from CrossBow Technology, though not recent, was chosen as one of the reference motes for comparison. A snapshot of typical wireless sensor nodes from a recently published research article is shown in Figure 2.

In what follows, a discussion about the recently proposed wireless sensor nodes published in the period from 2016 to 2022 and the commercially available wireless sensor nodes is provided. These nodes are categorized according to their potential applications such as: human monitoring and tracking, environmental monitoring and smart farming, smart cities, automotive applications, general IoT applications and seismic monitoring applications. The discussion focuses on the wireless sensor nodes’ applications, processing and memory specifications, communication capabilities, sensor support, power supply specifications and power consumption. 

### 3.1. Human Monitoring and Tracking

#### 3.1.1. InfiniTime

InfiniTime [18] is a wrist-wearable sensor node that was tailored to record images, audio, video, accelerometer data and temperature for tracking purposes. It utilizes a near-field communication/radio frequency identification (NFC/RFID) tag-integrated circuit (IC) with 16 kB electrically erasable programmable read-only memory (EEPROM) and inter-integrated circuit (I2C) bus interface. Regarding the node’s communication capabilities, wireless communication is facilitated by the M24LR16E-R transceiver manufactured by ST Microelectronics. The data rate is variable from 6.6 kbps to 26 kbps. Furthermore, the node contains an on-board ultra-low-power 3-axis accelerometer (ADXL362) by Analog Devices, temperature sensor, INMP801 MEMS analog microphone and an external 112 × 112 pixel analog camera. 

Concerning the node’s power supply specifications, the node is powered by solar and thermal energy harvesters. The solar panel consists of a parallel connection of eight small amorphous silicon solar cells (AM-1417CA) from Sanyo Energy. The thermal energy harnessing is facilitated by seven thermoelectric generator (TEG) modules. A single 40 mAh, 3.7 V lithium-polymer (LiPo) rechargeable battery is used for energy storage. Regarding the node’s power consumption, at 8 MHz MCU clock frequency, the current draw during ultra-sleep, idle, display update and data sending (radio only being active) modes is 1.225 µA, 859 µA, 4278 µA and 0.442 µA, respectively.

#### 3.1.2. Wireless Sensor Node with Application in Sports

The proposed low-cost sensor node finds applicability in sports to monitor athletic abilities such as reflexes and reaction speeds [19]. However, the nodes can be configured to suit other applications. The Arduino Pro Mini 3.3 V is used for the processing of sensed data. The board incorporates an Atmel ATmega328P microcontroller consisting of 32 kB of flash memory and an 8 MHz quartz crystal. Regarding its communication capabilities, the ultra-low power nRF24L01 transceiver is used for communication. It operates at 2.4 GHz using data rates of 250 kbps, 1 Mbps and 2 Mbps. The transmission power can be set to −18 dBm, −12 dBm, −16 dBm or 0 dBm. The transceiver is capable of achieving coverage of up to 250 m at a 250 kbps data rate and 0 dB transmission power.

Concerning the node’s capability to accommodate sensors, it is equipped with a connection interface consisting of 14 digital input/output pins and 6 analog inputs for facilitating connection to different sensors. The node can be powered by battery voltages ranging from 3.35 V to 12 V, thanks to the presence of the Arduino Pro Mini 3.3 V voltage regulator. A 3.7 V lithium ion (Li-ion) rechargeable battery, a 5 V rechargeable power bank or a 5 V, 5500 mAh Li-Po rechargeable battery can be utilized for power supply. The average current consumption in transmission and power-down modes is 5.4 mA and 0.5 mA, respectively.

### 3.2. Environmental Monitoring and Smart Farming

#### 3.2.1. MoleNet

The MoleNet [20] sensor node facilitates underground to underground (UG2UG) and underground to aboveground (UG2AG) communication for underground soil-monitoring applications. It was developed around the Wattuino Pro Mini, which exploits the Atmega328p microcontroller. The authors utilized an RV8523 real-time clock (RTC) for local time upkeep and removing the MCU from deep sleep mode. It offers the capability of operating in six sleep modes. 

Regarding the MoleNet’s communication capabilities, a HopeRF RFM69CW transceiver was chosen for UG2UG and UG2AG communication. At 433 MHz transmission frequency, an UG2AG transmission range of 80 m was obtained, while a 20 m communication range was yielded by the 868 MHz transceiver. An UG2UG communication range of 7.5 m is achievable using the transceiver at 433 MHz. The proposed node possesses on-board volumetric water content and temperature sensors for underground deployment. 

A battery life of nearly 5 years is reported by the authors. For power supply regulation, a power-efficient MCP1703 regulator is used. Power consumption is approximately 19 mA during sensing, 56 mA during transmitting and 5 mA in idle states.

#### 3.2.2. Sensor Nodes for Agriculture and Rural Monitoring

The sensing platforms proposed by [21] are targeted for rural monitoring and agriculture applications. The STM32L152 MCU with 128 kB Flash, 16 kB static random-access memory (SRAM) and 4 kB electrically erasable programmable read-only memory (EEPROM) was incorporated in the sensor modules. However, the specifications of the MCU have since changed. The ultra-low-power 32-bit MCU Arm^®^-based Cortex^®^-M3 (32 MHz) now comprises 512 kB Flash memory, 80 kB SRAM and 16 kB EEPROM. Regarding communication capabilities, the modules are equipped with IEEE Std. 802.15.4™-compliant RF transceiver wireless modules (MRF24J40) capable of covering ranges above 120 m in outdoor scenarios. The transceivers operate in the unlicensed ISM Band (2.405 GHz–2.475 GHz) and can achieve up to 500 kbps data rates. The receiver sensitivity of the modules is about −94 dBm. 

The sensing modules are integrated with SHT11 (air temperature and humidity) and 5TM (soil temperature and moisture content) sensors. The node offers the flexibility of accommodating other types of commercial sensors. The power supply required for the modules ranges from 2.7 to 3.3 V. The current consumption during the receiving mode is 20.2 mA and 19.7 mA during transmission mode at 0 dBm transmission power. 

#### 3.2.3. FROG Node

The Fog-computing platform “FROG” [22] was developed for smart city and precision agriculture applications. The FROG node is based on the Arduino Fio V3 that relies on the low-power ATmega32U4 microcontroller having 32 kB flash program memory, 2.5 kB SRAM, 1 kB EEPROM. A throughput of 16 million instructions per second (MIPS) at 16 MHz is attainable to allow for power consumption optimization against processing speed. An XBee-PRO XSC radio system that operates in the 902–928 MHz band was chosen for communication. A software-selectable transmit power output of 24 dBm is achievable by the radio system. The receiver sensitivity is −109 dBm at 9600 band. The ideal line-of-sight communication range can reach up to 45 km.

The node is equipped with several on-board gas sensors including CO_2_ sensors. A 12 V Li-Po battery with a capacity of 6400 mAh is utilized for powering the sensor node. Regarding power consumption, the FROG node’s processor and the sensors altogether draw 741.98 mA of current. The current consumption of the XBee-PRO XSC radio module is around 266.5 mA.

#### 3.2.4. Sensor Node Platform for Agriculture Monitoring 

The node proposed by [23] was explicitly designed for agriculture-monitoring applications. The module is built around an ATmega328P-PU microcontroller with 2 kB RAM, 1 kB EEPROM and 32 kB in-system self-programmable flash memory. It can achieve 16 MIPS at 16 MHz clock speed. Communication by the proposed node was realized using the CC1101 transceiver from Texas Instruments operating in the 300–348 MHz, 387–464 MHz and 779–928 MHz frequency bands. In the 868/915 MHz band, it is capable of transmitting at a maximum power of 12 dBm. The data rate is adjustable from 0.6 to 600 kbps. Communication ranges of over 400 m are achievable by the node. The hardware was developed around a panStamp NRG2 module.

An on-board Bosch Sensortec BME280 environmental sensor is utilized for measuring temperature, humidity and air pressure. The proposed mote can accommodate additional sensors for agriculture-monitoring purposes. The node is powered by a series connection of two standard AAA alkaline batteries that supply 3 V. Energy harvesting systems can be easily incorporated to the mote. Regarding power consumption, the sensor mote consumes 1.1 µA during sleep mode. The average current consumption during transmission is approximately 17.5 mA at 0 dBm and around 30.1 mA at 12 dBm. 

#### 3.2.5. Wireless Sensor Networks for Critical Event Detection 

The sensor node proposed by [24] was developed for environmental monitoring and fire detection. It was designed around Libelium’s Waspmote microcontrollers (ATmega1281) operating at 8 MHz with 8 kB SRAM, 4 kB EEPROM, 128 kB FLASH and 2 GB of SD card memory. A hybrid of communication modules was utilized. The authors opted for the limited range XBee S1 Pro operating at 2.4 GHz band and the wide range XBee 900LP operating at the 900 MHz band. Furthermore, the humidity, carbon monoxide, temperature and pressure sensors were exploited by the authors. The node relies on a rechargeable battery for power supply. There is an option for a solar panel to harvest energy. The power consumption of the node was not specified by the authors.

#### 3.2.6. “The Smaller the Better” Sensor Node 

The node proposed by [25] was designed for the long-range monitoring of environmental parameters such as temperature and humidity. Regarding the processor and memory, the CMWX1ZZABZ-078 chip by ABZ Murata that integrates a microcontroller (STM32L082CZ), a low-power long-range (LoRa) module (SX1276) and an impedance matching line on a single system-on-chip was utilized by the authors. The STM32 micro-controller is a Cortex M0+ type containing 192 kB flash memory, 20 kB SRAM and 6 kB EEPROM.

The LoRa module capable of transmitting at 868 MHz and 915 MHz was chosen for communication. The module’s output power is around 14 dBm. Furthermore, the nodes are equipped with an on-board temperature sensor, humidity sensor and a pluviometer for measuring rain precipitation. Power is supplied by energy harvesters (solar panel, Peltier module). The harnessed energy is stored by a supercapacitor and/or a battery. An energy manager chip (SPV1050) and a chip for monitoring the battery current and voltage are also incorporated in the nodes. The LoRa transceiver consumes 0.5 µA in the sleep state and 41 mA at 14 dBm transmission power while the MCU consumes 2.8 µA in the sleep state and 9.5 mA in the active state.

#### 3.2.7. Wireless Multi-Sensor Node

The multi-sensor node [26] was designed to cater for monitoring environmental parameters. The node was developed around the STM32F103ZET6 MCU, which is based on the ARM^®^ Cortex^®^-M3 core operating at a 72 MHz frequency. It contains up to 512 kB Flash memory and up to 64 kB SRAM. The authors utilized the wireless fidelity (Wi-Fi) to link a gateway with the sensor motes at 2.4 GHz. The ESP8266 transceiver module is exploited for communication. The node accommodates UV light (GT-302), raindrop, noise and SM300D2 seven-in-one (temperature, humidity, PM2.5, PM10, formaldehyde concentration, CO_2_ concentration and TVOC concentration) sensors to measure environmental parameters.

The node is powered by a battery and a solar panel connected to a solar tracking system. Regarding the power consumption, the mote consumes around 0.65 W during sensing. The current consumption by the Wi-Fi during the transmission mode is approximately 100 mA. The sensor node consumes around 0.8 W of power in the transmission mode. During the sleep state, the current consumption is about 50 mA due to the STM32 core plus 10 mA because of other standby circuits. Approximately 0.3 W power is consumed by the mote in the sleep state.

#### 3.2.8. Airborne Sensor Motes

The airborne sensor motes [27] were tailored for atmospheric sensing applications. The nodes are endowed with a Texas Instruments microcontroller (CC430F5137) that is composed of 32 kB of programmable flash memory and 4 kB of RAM. The microcontroller combines the advantages of the features of the CC1101 sub-1 GHz RF radio module with the MSP430 CPUXV2. The MSP430 CPU has a 16-bit reduced instruction set computer (RISC) architecture with a clocking frequency of up to 20 MHz. 

The proposed nodes can communicate using 130 individual channels containing 16 time slots per second by exploiting the industrial scientific and medical (ISM) band (915 MHz) transceivers. A u-Blox Max M8 GPS receiver was also utilized for communication. A multiple frequency time-division multiple access (MF-TDMA) technique is used to facilitate the reception of data from several eMotes at once. A communication range of up to around 50 km is realized by the node. Moreover, the temperature and air pressure sensor (MS5803) and the Sensirion humidity and temperature sensor (SHT25) can be accommodated in the proposed nodes. Power supply and power consumption details were not specified by the authors.

#### 3.2.9. Wildlife Observation Sensor Node

The node presented by [28] was mainly developed for wildlife (chimpanzee) monitoring purposes. Regarding processor and memory specifications, a CY8C4248LQI-BL583 module by Cypress that incorporates a 32-Bit Arm Cortex-M0 CPU with 256 kB of flash and 32 kB of SRAM was exploited by the authors. The module can be interfaced with a 32 GB SD card module for providing extra memory for measured data. 

The CY8C4248LQI-BL583 module by Cypress that incorporates a Bluetooth low-energy (BLE) module was chosen for communication at 2.4 GHz. The RF output power ranges from −18 dBm to +3 dBm. No sensors were incorporated in the node. Instead, it relies on the received signal strength indicator (RSSI) value to compute the distance between the animals. A 2600 mAh, 3.6 V lithium thionyI chloride battery is utilized to supply power to the node. At optimized performance, the BLE module draws a 1.9 mA current and consumes 4.99 mW of power. At 46 MHz, the CPU draws 27.7 mA. When the processor is inactive, it draws 1.7 mA and consumes 1.5 µA of current when it goes to the deep sleep mode.

#### 3.2.10. Smart Helmet

The Smart Helmet [29] node is targeted for air quality monitoring in the mining industry. The Adafruit LoRa feather board with an inbuilt 48 MHz ARM Cortex M0+ (SAMD21) microcontroller by Microchip was chosen for the node. It contains 256 kB flash memory and 32 kB of static RAM. A LoRa module (RFM69) embedded in the Adafruit LoRa feather board was used for communication. It is capable of operating in the 868 MHz/915 MHz/865 to 867 MHz/923 MHz unlicensed bands in different countries. The module is capable of producing an RF output of +20 dBm. A communication range of around 500 m LOS is also achievable.

A SparkFun SEN-16531 air quality sensor (SGP30) is incorporated for measuring levels of carbon dioxide and total volatile organic compound (TVOC). A battery is used to supply power to the node and the power consumption details are not specified.

#### 3.2.11. Drone-to-Sensor Wireless Ranging Platform

The platform for unmanned aerial vehicles (UAVs) proposed by [30] is targeted for environmental applications requiring mobility and other applications that require drone-to-wireless sensor systems. Regarding the processor and memory specifications, the STM32WB55RG MCU was utilized by the authors. The MCU consists of the ARM Cortex-M4, which is used for the central processing tasks, and the ARM Cortex-M0 to cater for the radio communication protocols. It comprises up to 1 MB of embedded flash memory available for storing programs and data and 256 kB of partitioned SRAMs. 

The IEEE 802.15.4-2011-compliant Decawave (DW1000) transceiver was utilized. It supports frequency bands with center frequencies ranging from 3.5 GHz to 6.5 GHz. It is capable of achieving communication ranges of up to 300 m. The platform incorporates on-board magnetometer, temperature, pressure and humidity sensors. External sensors are also supported. Supercapacitors/batteries are used to power the WSN platform. Energy harvesting using inductive power transfer is also proposed by the authors. The sensor node consumes 190 µW of power in the sleep state and 29 J of energy is consumed during the landing stage of the drone.

#### 3.2.12. Smart Wireless Climate Sensor Node

The proposed smart wireless climate sensor node [30] was designed for indoor comfort quality-monitoring applications. Pertaining to the processor and memory specifications, the node was developed around the ATMEGA-4808 microcontroller with 48 kB of flash memory and 256 bytes of EEPROM. The Wi-Fi module (WINC15100) was utilized for communication purposes. 

The node supports an SHT sensor module that is capable of measuring temperature and relative humidity levels. It is powered by either a supercapacitor or rechargeable Li-ion battery. A battery charger unit is also incorporated. Concerning the power consumption, the sensor node consumes 755.86 mW of power when transmitting both temperature and humidity, and 94.65 mW in the low-power mode.

### 3.3. Smart Cities and Automotive Applications

#### 3.3.1. Sensor Nodes for Smart Cities

The sensor nodes proposed by [13] were developed for smart cities and remote sensing applications. Pertaining to the processor and memory specifications, the nodes were designed around the 8 MHz Waspmote microcontroller (ATmega1281) consisting of 8 kB SRAM, 4 kB EEPROM, 128 kB FLASH and 2 GB SD card memory. Two ZigBee radio modules are utilized for realizing communication between the sensor motes. A 2.4 GHz ZigBee module (XBee-PRO version) is chosen for communication between ground nodes and the other ZigBee module operating at 868 MHz is used for linking with the UAV node. A radio expansion interface is used for facilitating a node to simultaneously connect to two different ZigBee transceivers. The 2.4 GHz XBee-PRO can operate at a transmission power of 18 dBm with sensitivity of −100 dBm while achieving a communication range of 1500 m. The 2.4 GHz XBee S2 version can transmit at 3 dBm power with a receiver sensitivity of around −97 dBm covering up to 120 m. 

The sensor nodes utilize external sensor boards with sockets for accommodating the HC ultrasonic sensor and the DHT11 sensor (temperature and humidity), Grove-Gas Sensor (MQ2) (for detecting the leakage of gases that include hydrogen, liquefied petroleum gas (LPG), methane, CO, alcohol, smoke or propane) and the D-Sun Hc-Sr501 pyroelectric infrared (PIR) motion sensor. In addition, external sockets are available for connecting new types of sensors. Batteries were utilized to power the nodes during the first implementation of a WSN by the authors. During the second implementation, the Waspmote board that can accommodate a solar panel was utilized. The low power consumption during data communication was realized using ZigBee modules.

#### 3.3.2. Wireless Sensor Network Hardware for Automotive Applications

The WSN node proposed by [31] was developed for intelligent automotive applications targeting public transportation. It focuses on monitoring mechanical and environmental stresses experienced by a travelling bus and its passengers. The node was developed around a Raspberry Pi 3 Bi+, which is equipped with a high-speed (1.4 GHz) processor (64-bit Cortex-A53) containing 1 GB RAM and up to 64 GB disk storage.

Regarding the node’s communication capabilities, the Huawei E3372 Megafon dongle was adopted as the communication module. It operates in the global system for mobile (GSM) communication (2G, 900 and 1900 MHz), universal mobile telecommunications system (UMTS) (3G, 900 and 2100 MHz), and long-term evolution (LTE) (4G, 800, 1800, 2100 and 2600 MHz) frequency bands. It is capable of operating at data rates from about 236.8 kbps to 150 Mbps. Furthermore, the node consists of on-board accelerometric, temperature and relative humidity sensors. The capacitive sensor (SNS-DH11) was chosen to monitor relative humidity readings varying from around 1% to 90%, with a precision of about ±5%. The temperature sensor (Ad-001 Ds18b20) was selected to measure temperature readings. A 3-axis accelerometer ADXL-345 (SparqEE LLC, Placentia, CA, USA), based on the MMA7361 device, is utilized to detect mechanical stresses. 

Concerning the power supply of the node, the B1700526EU (Yeeco, Germany) DC-DC converter is utilized to down-convert the 24 V supplied by the vehicle battery to the 5 V needed to power the Raspberry Pi. A second DC-DC converter (ZC104300 CPT) is also incorporated to supply 12 V to a monitor. The proposed node consumes approximately 0.08 kWh at peak. Since the node utilizes the vehicle’s battery (~243 kWh) for power supply, it means that the energy efficiency of this node is not a critical factor. 

#### 3.3.3. Plant Microbial Fuel Cell-Based Wireless Sensor Node

The self-powered plant microbial fuel cell (PMFC)-based wireless sensor node [32] is targeted for monitoring the environment of smart cities. Concerning the processor and memory specifications, the 16-bit RISC ultra-low-power MCU (MSP430FR5969) developed by Texas Instruments contains 64 kB ferroelectric random-access memory (FRAM) and can attain a clock speed of 16 MHz was utilized.

The LoRa radio module (RFM95W), which utilizes the SX1276 LoRa transceiver with serial peripheral interface (SPI), was adopted by the authors. The transceiver operates in the 868.1 MHz band with a coverage range of 2 km in line-of-sight situations or approximately 20 km using directional antennas. The transmit power is adjustable ranging from around +5 to +20 dBm. Furthermore, the MQ-131 and MQ-135 sensors are exploited for detecting ozone and carbon dioxide levels, respectively. 

The node is powered by energy harvesting using PMFCs. The PMFCs are capable of supplying 1.25 $\mathrm{mW}/{\mathrm{cm}}^{2}$ to the energy-scavenging circuit (BQ25570), which supplies a voltage of around 3.3 V. A supercapacitor is used for energy storage. The MCU (MSP430FR5969) can operate in the standby low-power mode (LPM3) and real-time clock (LPM3.5) mode, having typical current consumptions of approximately 400 nA and 250 nA, respectively. The peak current at maximum power output (20 dBm) is approximately 30 mA when the transceiver is in the active-listening state. The average power consumption of the mote is 2.92 mW.

### 3.4. General IoT Applications

#### 3.4.1. MEGAN

MEGAN [14] is a sensor node that serves a variety of IoT applications. The low-power, advanced RISC-based ATmega324PA microcontroller was utilized. The microcontroller contains 32 kB flash, 2.5 kB static RAM, and 1 kB of EEPROM. A clocking frequency of 8 MHz was utilized by the authors but can be increased to 16 MHz. The mote offers the advantage of choosing a communication module for a particular application. In their demonstration, the authors utilized ZigBee (XBee series 1) and Bluetooth (HC-05) modules for communication. However, according to the authors, the node can support other communication technologies such as general packet radio service (GPRS), Wi-Fi, and the GSM. Besides, the node consists of 32 general purpose input/output (GPIO) pins that enable several sensors and actuators to be connected to it. 

A 3.7 V, 800 mAh Li-ion battery is utilized to power the node. The battery is recharged using a recharging circuit incorporated in the sensor mote. The current consumption of the node in the sleep and active states is 10.1 µA and 7.95 mA, respectively. The consumption in the active state when utilizing the Bluetooth module is 16.44 mA, while it is 59.65 mA when the Zigbee module is selected.

#### 3.4.2. WaterGrid-Sense

WaterGrid-Sense [33] is a LoRa-based sensor mote targeting the IoT in industrial set-ups, especially smart water management systems. The availability of sensor interfaces enables its usage in different applications. The processor and memory specifications of the mote are not specified by the authors. The LoRa radio module operating at 868 MHz was adopted for long-range communication. In addition, the WaterGrid-Sense mote houses the pressure and pulse sensor interfaces. The pressure sensor is installed on a water pipe while the pulse sensor is installed on a water meter. The pressure and water meter readings are transmitted to users via the LoRa gateway. 

Concerning the mote’s power supply, it relies on a 1000 mAh, 3.7 V Li-ion battery and is capable of scavenging solar energy by means of an external solar panel. The charge limit of the battery is 4.2 V at 500 mA. The node consumes 0.1 mV, 23 mV and 73 mV in the sleep, sensing and the transmission states, respectively. The node draws approximately 0.52 mA, 0.16 mA, 0.1 mA during the transmission, in idle and sleep states, respectively. The power consumed during transmission, sensing/idle and sleep states is around 190 mW, 52 mW and 0.1 mW, respectively. On average, a battery voltage of around 0.067 V is consumed per day. 

#### 3.4.3. Wireless Sensor Node for IoT

The wireless sensor node developed by [34] is targeted for IoT-based applications. Regarding the processor and memory, the node is equipped with an ATMega88 microcontroller containing 8 kB flash memory and 1 kB SRAM. The BLE and nRF24 L01 RF (2.4 GHz) transceivers were utilized for communication. The communication ranges for the two transceivers are 10–30 m and 400–1000 m. The nRF24 L01 wireless module and the BLE transceiver (HM10BLE) have data rates of 2 Mbps and 3 Mbps, respectively. The node houses temperature, humidity and current sensors.

The node relies on both the Li-ion battery (3.6 V to 4.2 V) and supercapacitor for power supply. The current consumption of the RF transceiver (nRF24 L01) is 7–11.3 mA during the transmission mode, 9–13.5 mA during receiving mode, 26 µA during the sleep mode and 18 mA is the peak current. The transceiver’s power consumption is 60 mW. The BLE communication module consumes 8.5–9 mA during the transmission and receiving modes, 0.78 µA in the sleep state, 15 mA peak current. Its power consumption is 10 mW.

#### 3.4.4. NanoICARUS Mote

The nanoICARUS mote [35] was designed for IoT applications. Concerning the processor and memory specifications, the mote incorporates the ultra-low power ARM Cortex-M4 32-bit RISC core MCU (STM32L476RG) that can operate at up to 80 MHz. It contains 1 MB flash memory and static RAM of 128 kB. Also, an off-chip FRAM (MR45V256A) is installed on the board for optional memory. Pertaining to the communication capabilities, the authors utilized an XBee footprint socket for accommodating radio modules such as LoRa, ZigBee, BLE, etc. In addition, the temperature and humidity sensor (SHT21), light intensity sensor (VEML6030) and the battery gauge (MAX17048G+) are integrated in the prototype node. An option for additional sensing modules is available through the I2C and I/O ports. The sensor node can be powered by voltages ranging roughly from 1.5 V to 3.7 V. A current of around 33 nA is consumed during the sleep state due to the presence of an off-chip RTC and power switch. The mote consumes around 14 mA in the active state.

#### 3.4.5. Flexible Fog Computing-Based Sensor Node

The node based on flexible Fog computing [36] offers flexibility since it can be used in several IoT applications such as smart buildings, smart factories and smart ports. Regarding the processor and memory specifications, the node utilizes an Arm^®^ Cortex^®^-M4 MCU (CC3220MODASF12) at 80 MHz. It contains 256 kB of RAM and 1 MB flash memory for accommodating data and configuration parameters in the mote. 

The LoRa transceiver (RN2483) and Wi-Fi module (CC3220MODASF12MONR) are utilized to provide the wireless link for the nodes. The Wi-Fi module operating at 2.4 GHz is chosen for short-range (up to 100 m) communication while the LoRa transceiver is selected for long-range coverage of the order of kilometers. In addition, the node consists of an on-board three-axis accelerometer (ADXL355), a pressure/humidity/temperature sensor (BME280) and an inductance sensor (LDC1000) for detecting corrosion levels.

The node relies on a 3.6 V and 5800 mAh battery for power supply. Concerning the power consumption, at 3.3 V, the LoRa module consumes 420 mA while the system-on- module (SOM) draws 800 mA. With a battery capacity of 5800 mA/h, an approximately 1.7 year lifetime of the sensor node is reported.

#### 3.4.6. MICAz Mote

MICAz mote (MEMSIC) is one of the popular commercial wireless sensor motes. It was developed for applications that include indoor building monitoring and security, acoustic, video, vibration and other high-speed sensor data and large-scale sensor networks. Regarding the processor and memory specifications, the MPR2400 that is based on the Atmel ATmega128L low-power microcontroller was utilized. It comprises 128 kB program flash memory, 512 kB measurement flash memory and 4 kB of EEPROM for configurations. Communication between the motes is accomplished using the IEEE 802.15.4-compliant MICAz radio (MPR2400). It is capable of operating within the 2.40 GHz–2.48 GHz band at up to 250 kbps. The RF transceiver’s output power ranges from −24 dBm to 0 dBm. In outdoor and indoor scenarios, communication ranges of up to 100 m and up to 30 m, respectively, are achievable. 

The mote can accommodate a number of different sensor and data acquisition boards through a standard 51-pin expansion connector. In addition, custom sensor boards are also offered. Concerning the power supply, the mote can be powered by two AA batteries. It requires 2.7 V–3.3 V power to operate. The RF transceiver draws approximately 17.4 mA of current when sending data at a power of 0 dBm, 1 µA in the sleep mode and 20 µA in the idle mode. The processor board draws about 8 mA of current in the active mode and below 15 µA in the sleep mode.

#### 3.4.7. IRIS 

The IRIS mote (MEMSIC) is a popular commercial mote developed for applications that include indoor building monitoring and security, acoustic, video, vibration and other high-speed sensor data and large-scale sensor networks. The mote utilizes the XM2110CA processor, which is based on the Atmel ATmega 128-1, with 8 kB RAM, 128 kB program flash memory and 512 kB measurement flash memory, and 4 kB of configuration EEPROM. The IEEE 802.15.4-compliant RF transceiver operating in the 2.405 GHz to 2.480 GHz ISM band was chosen for the IRIS mote. The radio can transmit at data rates of up to 250 kbps at a power of 3 dBm. The indoor coverage range is more than 30 m and can reach above 300 m outdoors in line-of-sight situations. 

Several sensor and data acquisition boards (including light, temperature, RH, barometric pressure, acceleration/seismic, acoustic and magnetic) can be connected to the platform thanks to the presence of the standard 51-pin expansion connector that facilitates plug and play installation. The mote requires 2.7 to 3.3 V power supply. A pack of two AA batteries is attached to the mote. Regarding the power consumption, the mote’s processor draws approximately 8 mA in the active mode and a total of about 3 µA in the sleep mode. Around 16 mA of current is drawn during the receive mode of the transceiver while 17 mA is drawn when transmitting at 3 dBm.

#### 3.4.8. LOTUS 

The applications of the popular commercial mote, LOTUS (MEMSIC), include seismic vibration monitoring, video, acoustic sensing and other applications requiring high-speed processing capability. The LOTUS mote is based on the low-power ARM Cortex M3 CPU containing 64 kB RAM, 512 kB program flash memory and the clock rate can reach 100 MHz. The radio module for LOTUS operates in the 2.40 GHz–2.48 GHz band at up to a 250 kbps data transmission rate. The outdoor coverage range is above 500 m. The mote does not possess on-board sensors but is capable of accommodating several sensor data acquisition boards. The LOTUS node can be powered by a 2.7–3.3 V power supply. Concerning the power consumption, the processor of the mote draws a current of about 10 µA during the sleep mode and around 50 mA in the active mode when operating at a 100 MHz clock rate. 

#### 3.4.9. WiSense

The WiSense module is another popular commercial mote that consists of two printed circuit boards (PCBs). One PCB houses the microcontroller, while the other houses the CC1120 radio. The mote is mainly targeted for very-low-power wireless applications including remote sensing. The module was built around a 16-bit ultra-low-power microcontroller (MSP430G2955) by Texas Instruments, containing 56 kB flash, 4 kB SRAM, 128 kB EEPROM and a 16 MHz clock. Regarding communication capabilities, a sub-1 GHz transceiver (CC1120) incorporated in a PCB separate from that of the microcontroller was utilized. It can communicate in the 865–867 MHz band and can achieve communication ranges of over 1 km line-of-sight at 1.2 kbps. The output power can reach up to +13 dBm. The data rate can vary from 0 to 200 kbps. In addition, an on-chip temperature sensor is accommodated in the sensor node.

The operating voltages of the nodes vary from 1.8 V to 3.6 V. A 3 V lithium coin cell or a series connection of two 1.5 V AA/AAA batteries can be used to power the nodes. A battery holder should be bought separately. An option is also available for a combination of a solar panel and a Li-ion battery for powering the mote. Concerning power consumption, the standby current (in LPM3) of the mote is as low as 1 µA.

#### 3.4.10. Waspmote Plug & Sense! 

The Waspmote Plug & Sense! mote by Libelium is also a popular commercial mote targeted for IoT applications. Pertaining to the processor and memory specifications, the mote is based on the Atmega 1281 MCU comprising 8 kB SRAM, 4 kB EEPROM (with 1 kB reserved), 128 kB Flash, 2 GB SD card and it can operate at 14.7456 MHz. The mote contains an internal SD card and external cards of up to 16 GB are supported. Regarding the communication capabilities, it can support short-, medium- and long-range communication technologies that include XBee-PRO 802.15.4, XBee-PRO DigiMesh, low-power wide area network (LPWAN) (Sigfox, LoRaWAN, LoRa), cellular (4G/3G/GPRS/GSM), Bluetooth PRO, Bluetooth low energy, near-field communication/radio frequency identification (NFC/RFID) 13.56 MHz and industrial protocols such as the recommended standard 232 (RS-232)/Modbus, the recommended standard 485 (RS-485)/Modbus and controller area network (CAN) bus. 

Waspmote Plug & Sense! consists of an in-built three-axis accelerometer and real-time clock temperature sensor. The node also incorporates a specific default socket for basic sensors that include the light-dependent resistor (LDR) and humidity and temperature sensors. The mote is capable of connecting a maximum of six sensor probes at a time. Each signal-conditioning circuit is dedicated for a certain model to facilitate the connection of specific additional sensors. The node can be powered by rechargeable lithium ion batteries (capacity of 6600 mAh) of battery voltages ranging from 3.3 to 4.2 V. It also offers an option for energy harvesting using an external solar panel. Concerning power consumption, the current consumed during the ON, sleep, deep-sleep and hibernate states is 17 mA, 30 μA, 33 μA and 7 μA, respectively.

### 3.5. Seismic Monitoring Applications

#### 3.5.1. Jennic

The piezo-based Jennic wireless sensor node [37] was explicitly designed for impact detection and the monitoring of buildings. Concerning the processor and memory specifications, the Jennic mote is built around a 16 MHz JN5139 microcontroller containing 96 kB RAM and 192 kB read-only memory (ROM). The JN5139 device is also capable of operating at 32 MHz. Its architecture is based on a 32-bit RISC CPU. Pertaining to the communication capabilities, a wireless transceiver that is compliant with the 2.4 GHz IEEE 802.15.4 protocol is incorporated in the node. A piezoelectric ceramic (PZT) is utilized for impact detection.

Regarding the power supply, a 9 V battery is needed for the wireless coordinator and a 3 V AA battery is required for the sensor. Dual voltage supplies are necessary in order for piezo-based applications to successfully perform sensing and actuating functions. The piezo-based sensor node consumes a maximum current of 37 mA at 3.6 V supply. The sleep current of the node is 2.6 µA when the sleep timer is ON.

#### 3.5.2. Wireless Sensor Node for Seismic Monitoring

The node proposed by [30] was developed for the seismic monitoring of buildings. Pertaining to the processor and memory specifications, the node was developed around the launch pad CC3200 ARM^®^ Cortex^®^-M4 Core-based microcontroller (at 80 MHz) by Texas Instruments. It contains up to 256 kB RAM and external serial flash bootloader, and peripheral drivers in ROM. The Wi-Fi incorporated in the CC3200 chip is used for communication among the sensor nodes. Furthermore, the node accommodates a three-component seismic sensor (Mark-l-4C3D) having a natural frequency of 1 Hz. The node can be powered by 5 V that can be obtained from batteries or a micro-USB charger. Concerning power consumption, the node draws 70 mA and 80 mA of current in the standby and sampling modes, respectively.

## 4. Technical Discussion

### 4.1. Mote Specifications and Applications

#### 4.1.1. Processor and Memory Capabilities

As can be observed from Table 1, the most widely used microcontrollers in the wireless sensor nodes considered in this paper from 2016 to 2022 are the Atmega series, which are targeted for low-power operation.

Generally, regarding the RAM, Flash and EEPROM capacities, most of these sensor nodes and also commercial motes reviewed in this paper have comparable memory that is limited. Hence, they are most suitable for applications that are not data intensive. It is also noted that some of the motes, such as the Waspmote Plug & Sense! V15 utilize external SD cards with large storage capacities. However, writing to the SD cards is power-demanding and therefore limits the lifetime of the already power-constrained nodes. As can be seen in Table 1, from 2019 to 2022 the ARM Cortex series of MCUs have been dominating in most of the recent wireless sensor nodes reviewed in this paper. For example, the ICARUS [35] and that by [36] exploit the ARM Cortex-M4 32-bit RISC core MCU. The ARM Cortex series have the advantage of high-speed clock operation and improved memory capacity. Therefore, they are suitable for applications that are data intensive and require high-speed operation. Adjustable CPU clock rates permit a trade-off between power consumption and processing speed as guided by the WSN application requirements.

#### 4.1.2. Power Consumption

The Atmega328P has six sleep modes of operation to minimize power consumption. The JN5139 microcontroller consumes as much current in the active state as the other MCUs reviewed in Table 2 and Table 3. The CC430F5137 is capable of ultra-low power operation, since it consumes as little as 2.0 µA in standby mode (LPM3 RTC mode). 

It is also worth noting that the ICARUS mote that utilizes the STM32L476RG MCU can consume as little as 22 nA during the sleep state. The STM32L476RG MCU and ARM Cortex-M4 MCU-CC3200 in the ICARUS mote have the advantage of operating at up to 80 MHz clock frequency. However, utilizing high-speed and powerful processors leads to increased energy consumption and cost. Therefore, it is necessary to optimize power consumption versus processing speed. The ATmega1281 MCU is appropriate for applications that do not require high clock frequency operation. In order to minimize the power consumption in WSNs, dynamic power management techniques including dynamic scaling, duty cycling, data aggregation, data prediction and data compression should be implemented and improved.

#### 4.1.3. Applications

Most of the recent sensor nodes reviewed in this paper are targeted for environmental monitoring and various IoT applications. A notable number of the research nodes are designed for multiple applications to allow for flexibility. It can be observed from Table 2 that, of the selected commercial motes, only MICAz is targeted for multiple applications. LOTUS integrates the desirable features and characteristics of IRIS, TelosB and Imote2 motes onto a single board and can be used for applications demanding high processing speed thanks to the presence of the low-power ARM Cortex M3 CPU. Energy consumption by the sensor nodes also depends on the WSN applications. In some applications, it is possible to turn off some motes while other applications require all the motes to be always active.

Several applications and scenarios, including remote sensing, forest fire detection and smart parking systems, require sensor nodes capable of operating under ultra-low power consumption. In some of these applications, the motes are deployed in remote and inaccessible areas where it is difficult to replace or recharge depleted batteries. The NanoIcarus mote [35], Waspmote and the sensor node proposed by [25] are notable examples of nodes that are mostly optimized for power consumption preservation. For applications demanding high communication requirements, there is a need for motes that utilize efficient, long-range and low-energy-consumption communication modules. Applications that require highly accurate sensor measurements such as air quality monitoring and structural health monitoring need motes that have reliable hardware architecture and a communication system that has error correction/detection capabilities.

#### 4.1.4. Communication Capabilities

As can be observed in Table 2, several different types of transceivers have been utilized by the different sensor motes. The LoRa and ZigBee modules are the most commonly used transceivers by most of the recent sensor nodes. Sensor nodes such those by [14,24,33,35] and the Waspmote Plug and Sense! are capable of integrating different transceivers for communication. In order to minimize the overall sensor mote size, devices such as the Nordic nRF24E1 and ARM Cortex-M4 MCU-CC3200 integrate both the microcontroller and Wi-Fi (for internet connectivity) onto a single chip. Most of the radio modules operate at 2.4 GHz and are IEEE 802.15.4-compliant for interoperability purposes. The IEEE 802.15.4 communication protocol is suitable for low data rate WSN applications. The ZigBee protocol is chosen for its low power consumption. The Waspmote Plug & Sense! utilizes technologies such as Xbee-PRO and cellular networks to achieve wide coverage. However, in cellular networks there are much higher energy consumption and costs. LoRa transceivers have better energy efficiency and are also utilized for applications requiring long-range coverage. Introducing additional functionalities in a sensor node comes with an extra power consumption cost. In underground WSNs, network failure may be caused by high levels of signal attenuation. Appropriate and energy-efficient routing protocols should continue to be proposed to prevent premature overall network failure due to the nodes’ failure. Long-range and low-power communication modules should be developed to achieve the long network lifetime of WSNs.

#### 4.1.5. Sensor Support

Most of the commercially available sensor motes reviewed in this study do not offer all the basic built-in sensors. As an example, the Waspmote Plug & Sense! offers only two in-built sensors, namely the accelerometer and the temperature sensor. Instead, these commercial motes, such as the LOTUS, IRIS, MICAz and Waspmote Plug & Sense!, have external sensor boards with sockets to allow different sensors to be connected for flexibility. Some of the sensor node platforms also accommodate a range of GPIO ports. A notable number of sensor nodes from the recent research works provide built-in basic sensors.

#### 4.1.6. Power Supply

Most of the sensor nodes utilize lithium ion batteries and require battery voltages of up to around 3.7 V. A few sensor nodes from recent research articles such as those proposed by [25,32] and that by [24] utilized energy harvesting as an additional source of energy. Only the sensor nodes by [25,32] proposed the use of a supercapacitor for the storage of harvested energy. None of the popular commercial nodes reviewed in this paper considered the use of a supercapacitor for energy storage. Waspmote Plug & Sense! and Wisense are the only commercial motes reviewed in this paper offering an option for solar energy harvesting. It is also observed that, in all the articles reviewed in this paper, the authors did not introduce the use of a hybrid setup of energy harvesters for supplying additional energy to prolong the lifetime of the energy-limited wireless sensor nodes. However, it should also be noted that the size of the power supply system affects the overall physical size, cost and weight of the sensor node. Large-sized and heavy sensor nodes are not suitable for several application scenarios that require mobility and wearables such as wildlife monitoring and tracking and other health-monitoring applications.

#### 4.1.7. Hardware Security

Wireless sensor networks are targeted by hackers and attackers. Most of the sensor nodes reviewed in this paper do not include adequate security features, since the end devices are power-constrained. The nodes are vulnerable to various malicious attacks. The architecture of the node proposed by [35] is based on Fog computing to improve security and privacy. A pre-cloud link is introduced to safeguard the data. Transceivers such as the XBee modules offer advanced encryption standard (AES) encryption security features. Introducing other security algorithms incurs extra energy costs. Besides, the wireless sensor nodes have limited storage capacities to accommodate security algorithms. In an attempt to mitigate hardware security threats, ARM recently introduced the TrustZone technology to the microcontroller level (Cortex-M). The approach regards the hardware as the point of trust and then enables other resources of the sensor node to be trusted. Again, introducing additional security features is hindered by memory availability and energy constraints.

#### 4.1.8. Programming of Sensor Nodes

In this paper, it is noted that most of the sensor nodes are programmed using the standard languages (e.g., C/C++). The majority of the commercial motes utilize a generic, open-source programming environment. The software is developed using Arduino IDE as the base code environment and compiler. One of the key features to consider when designing sensor nodes is the operating system (OS) to utilize in the sensor motes. The OS significantly influences the stability, capability and security of the device. It should be easily upgradable without making several changes to the hardware. The open source TinyOS and Contiki are the most commonly utilized OSs. The commercially available Waspmote Plug & Sense! can be programmed using Libelium’s integrated development environment (IDE) that can be downloaded and installed on the Windows, macOS and Linux operating systems.

### 4.2. Challenges and Future Trends

The IoT technology utilizes WSNs for providing an efficient sensing and communication infrastructure. Despite numerous research efforts in WSNs, energy consumption remains a challenge that limits the lifetime of WSNs. Some of the critical features and characteristics that should be considered by designers of wireless sensor nodes are ultra-low power consumption, affordability, small physical size and software and hardware configurability. Microcontroller unit-based sensor motes have the advantages of low-power consumption, affordability, programming flexibility and fast time-to-market [9,38]. The nodes are mostly utilized for simple embedded systems with low budget requirements.

The general trend is to design and develop ultra-low power MCUs that consume ultra-low power when in the sleep state, since in most applications the sensor nodes are always in the sleep state. Also, the wakeup time of the motes must be reduced as much as possible. Therefore, advances in microprocessors and ultra-low power management techniques are critical to minimize power consumption and achieve the unlimited lifetime of WSNs. 

Low power MCUs are best suited for less computation-intensive applications. It can be observed that the recent wireless sensor nodes have started utilizing the ARM Cortex-based processors for applications requiring an increased computational power. Energy-harvesting techniques should be utilized for powering the wireless sensor node and extending the network lifetime. In order to achieve better energy efficiency and long-range coverage, the emerging LPWAN [39] technologies such as LoRa [40,41,42,43,44,45,46,47], NarrowBand IoT (NB-IoT) [48,49,50,51,52] and Sigfox [39] can be utilized. A combination of the advantages of these emerging and promising communication technologies also needs to be explored.

This study has revealed that most of the recent sensor nodes tend to follow an open source approach, while the older platforms follow a closed source approach. This is probably because using compatible open platforms and off-the-shelf components would allow researchers to rapidly develop improved sensor nodes in terms of power consumption, communication and processing capabilities as compared to the built-for-purpose platforms. Besides, software upgrades and changes on the hardware are made easier. However, relying on an open-source hardware setup does not potentially result in the reduction of the sensor node’s size. 

It is worth noting that ignoring the sensor node’s security issues and vulnerabilities during design compromises the functionality of the overall WSN. The dynamic or evolving nature of security threats calls for the need to continuously develop different mitigation technologies and countermeasures to guarantee end-to-end security in WSNs. Hence, there is a need for secure protocols that are computationally efficient and energy effective to match the limited resources of the motes. Reliable motes that give reliable measurements and do not often fail should be developed. These motes have to be robust against environmental changes by operating under different temperature, pressure, moisture and weather conditions without their accuracy being affected. The cost-effectiveness and portability of the motes are also crucial to facilitate easy deployment and utilization in many IoT applications. 

Exploring different means for energy harvesting, including wireless charging techniques that are both efficient and cost-effective, is still an open research area. How to manage the IoT motes remotely and update their firmware using technologies such as over-the-air programming (OTA) is an ongoing research. The energy efficiency issue affecting the implementation of UAVs or drones in applications requiring mobility also needs further research.

## 5. Conclusions and Future Work

In this paper, a detailed comparative review of MCU-based wireless sensor nodes from recently published research articles (2016–2022) has been presented. The sensor nodes from the recent research articles are compared with the most widely used commercially available motes based on processing and memory specifications, communication capabilities, power supply and consumption, sensor support, potential applications, node programming and hardware security. This paper attempts to provide a clear picture of the progress being made towards the design of energy-efficient and autonomous wireless sensor nodes to avoid redundancy in research by industry and research institutions. We anticipate this paper to assist developers of wireless sensor nodes to produce improved designs that outperform the existing motes. Besides, this paper guides researchers and potential users to easily make the best choice of a mote that best matches specific application scenarios. A discussion of the wireless sensor node platforms is provided. The challenges and future research directions are also outlined. The absence of real-field power consumption data for some of the sensor nodes reviewed in this paper can be considered as one of the limitations of this research. Ultra-low power techniques in wireless sensor nodes should continue to be explored. Miniaturization of the sensor nodes is also a future research direction that can be exploited. Security issues in sensor nodes must also not be ignored. We also recommend intensive experimental performance evaluations of the sensor nodes.

## Figures and Tables

**Table 1:** A summary of features and applications of sensor nodes from recent research articles.

Platform	CPU				Radio Transceiver			Tx Power	Applications
	Type	RAM	FLASH	EEPROM	Type	Freq	Range		
InfiniTime [18]	MSP430FR5969 microcontroller	64 kB (FRAM)	Not mentioned	Not mentioned	M24LR16E-R	400 kHz	Not mentioned	Not mentioned	Human tracking and monitoring
MoleNet [20]	Atmega328p microcontroller	2 kB (SRAM)	32 kB	1 kB and 25LC1024 1 MB EEPROM	RFM69CW	433 MHz/868 MHz	80 m	Not mentioned	Underground soil monitoring
[19]	Atmega328P microcontroller	2 kB	32 kB	1 kB	nRF24L01	2.4 GHz	250 m	−18, −12, 16 or 0 dBm	Sports (monitoring athletic abilities)
[21]	STM32L152 MCU	16 kB SRAM	128 kB	4 kB	MRF24J40	2.405 GHz–2.475 GHz	Above 120 m	Not mentioned	Rural monitoring and precision agriculture
[37]	JN5139 microcontroller	96 kB RAM	Not mentioned	192 kB ROM	IEEE 802.15.4 compliant	2.4 GHz	Not mentioned	3 dBm	Impact detection, SHM and wireless structural vibration control
[23]	Atmega328P-PU microcontroller	2 kB	32 kB	1 kB	CC1101 transceiver from Texas Instruments	300–348 MHz, 387–464 MHz and 779–928 MHz	Over 400 m	12 dBm	Agriculture monitoring
FROG [22]	Atmega32U4 microcontroller	2.5 kB SRAM	32 kB	1 kB EEPROM	XBee-PRO XSC radio system	902–928 MHz	45 km LOS	24 dBm	Smart cities and smart farming
[31]	64-bit Cortex-A53	1 GB RAM and up to 64 GB disk storage.	Not mentioned	Not mentioned	Huawei E3372 Megafon dongle	900/1900 MHz, 900/2100 MHz, 800/1800/2100/2600 MHz	Not mentioned	Not mentioned	Automotive
MEGAN [14]	Atmega324PA microcontroller	2 kB SRAM	32 kB	1 kB EEPROM	XBee series 1/Bluetooth (HC-05)/GPRS, Wi-F/GSM	2.4 GHz	Not mentioned	Not mentioned	Multiple IoT
[34]	ATMega88 microcontroller	1 kB SRAM	8 kB	512 kB	RFM transceiver (nRF24 L01) and BLE transceiver (HM10BLE)	2.4 GHz	10–30 m (BLE), 400–1000 m (RFM)	Not mentioned	IoT-based
[24]	ATmega1281	8 kB SRAM	128 kB FLASH	4 kB EEPROM	XBee S1 Pro and XBee 900LP	2.4 GHz, 900 MHz	Not mentioned	Not mentioned	Environmental monitoring and critical event detection
[32]	MSP430FR5969	64 kB non-volatile FRAM	Not mentioned	Not mentioned	LoRa module (RFM95W)	868.1 MHz unlicensed band	2 km in LOS or 20 km with directional antennas	+5 to +20 dBm	Smart cities
[28]	32-Bit Arm Cortex-M0 CPU	32 kB SRAM	256 kB flash	Not mentioned	BLE module	2.4 GHz	Not mentioned	−18 dBm to +3 dBm	Wildlife monitoring
[36]	Arm^®^ Cortex^®^-M4 MCU (CC3220MODASF12)	256 kB	1 MB flash	Not mentioned	LoRa (RN2483) and Wi-Fi modules	2.4 GHz	Not mentioned	Not mentioned	IoT
[29]	Arm cortex M0+ (SAMD21)	32 kB SRAM	256 kB flash	Not mentioned	LoRa (RFM69) module	868 MHz/915 MHz/865 to 867 MHz/923 MHz	500 m LOS	+20 dBm	Air quality monitoring in mining industries
[13]	ATmega1281	8 kB	128 kB	Not mentioned	ZigBee module (XBee-PRO and XBee S2)	2.4 GHz	1500 m (XBee-PRO), 120 m (XBee S2)	18 dBm (XBee-PRO), 3 dBm (XBee S2)	Multi-applications in smart cities and remote monitoring and sensing
[30]	ARM Cortex-M4 MCU-CC3200	256 kB	Not mentioned	Not mentioned	Wi-Fi-CC3200	Not mentioned	Not mentioned	18 dBm	Seismic monitoring of buildings

**Table 2:** A Summary of features and applications of widely used selected commercial motes.

Platform	CPU				Radio Transceiver			Tx Power	Applications
	Type	RAM	FLASH	EEPROM	Type	Freq	Range		
MICAz (2004)	Atmega128	4	128	512	CC2420	900	12	89 mW	Multi-applications
IRIS	ATmega 128-1	8 kB	128 kB + 512 kB serial	4 kB	IEEE 802.15.4 compliant	2.405 GHz to 2.480 GHz	>30 m indoor, >300 m outdoor	3 dBm	Indoor building monitoring and security, acoustic, video, large-scale sensor networks
LOTUS	NXP LPC1758 ARM Cortex M3 CPU	64 kB	512 kB	-	RF231	2.405 GHz to 2.480 GHz	>500 m		seismic vibration monitoring, video, acoustic sensing and high-speed processing
WiSense	MSP430G2955 by TI	4 kB SRAM	56 kB	128 kB	CC1120	865–867 MHz	>1 km	13 dBm	Very low-power including remote sensing
Waspmote Plug & Sense!V15	ATmega 1281	SRAM 8 kB,	Flash 128 kB, SD card	EEPROM 4 kB (1 kB reserved)	XBee-PRO, LPWAN, cellular, Bluetooth PRO, BLE, etc.	Wide range	short, medium and long range		IoT

**Table 3:** Power consumption of different MCUs.

MCU	Clock Freq. (MHz)	Power Consumption	
		Active	Sleep
Atmega328p	16	14 mA	66 µA
STM32L152 MCU	32	6.24 mA	4.6 µA
JN5139 microcontroller	32	37 mA	2.6 µA
Atmega324PA	20	0.4 mA@1 MHz, 1.8 V	0.6 µA (RTC on)
ATMega88	20	0.3 mA	0.8 μA (RTC on)
MSP430FR5969	16	103 µA/MHz	0.25 µA (LPM3.5)
STM32L476RG	80	100 µA/MHz	420 nA *Standby* mode (RTC on)
CC430F5137	20	160 µA/MHz	2 µA
Arm Cortex M0+ (SAMD21)	48	~7 mA	~12.8 µA
ARM Cortex-M4 MCU	80	229 mA	250 μA (LPDS), 4 μA (hibernate)
ATmega1281	8	500 µA/MHz	0.1 µA @1.8 V
ARM Cortex M3 CPU	120	195 µA/MHz	1.11 µA (RTC on)

## Data Availability

Publicly available datasets were analyzed in this study.
